# Celebrating the impact of advanced practice providers (APPs) in gastroenterology: honoring APP week 2025!

**DOI:** 10.1016/j.igie.2025.07.003

**Published:** 2025-07-05

**Authors:** Sarah Enslin, Alice Saji, Vivek Kaul

**Affiliations:** 1 Division of Gastroenterology and Hepatology, University of Rochester Medical Center, Rochester, New York, USA; 2 Department of Gastroenterology, Hepatology & Nutrition, The University of Texas MD Anderson Cancer Center, Houston, Texas, USA

## Introduction

Advanced practice providers (APPs), encompassing both nurse practitioners (NPs) and physician assistants (PAs), are integral members of the gastroenterology care team. As we celebrate National APP Week September 22 to 26, 2025, it is an ideal opportunity to recognize their multifaceted contributions in expanding access to gastroenterology services and elevating patient care.

In an ever-evolving health care landscape, the demand for high-quality, accessible, and efficient care continues to grow—especially in complex specialties like gastroenterology. From managing chronic diseases to helping support advanced endoscopy practices, APPs meet this demand by blending clinical acumen with patient-centered care. Working in close collaboration with physicians, many APPs also manage their own patient panels with a high degree of autonomy.

### Comprehensive clinical care

The integration of APPs into gastroenterology practices varies depending on practice needs, geographic location, and scope of practice as defined by state licensure. Their roles may encompass ambulatory care, inpatient care, or a hybrid model. APPs evaluate and manage a wide spectrum of gastrointestinal conditions, including inflammatory bowel disease, liver disease, motility disorders, gastrointestinal cancers, and therapy-related gastrointestinal toxicities.

**Figure 1:**
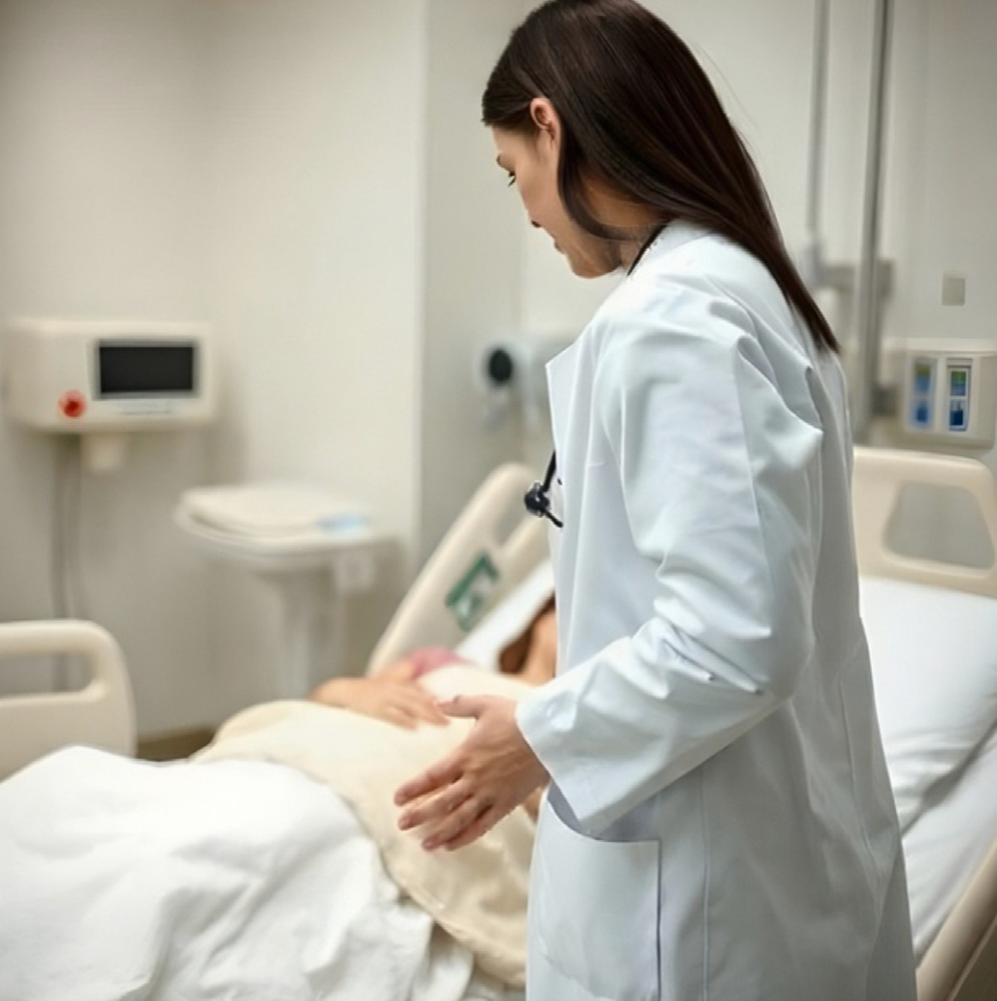
An advanced practice provider (APP) delivering hands-on bedside care, highlighting the essential role of APPs in direct patient care.

APPs are skilled in conducting comprehensive assessments, ordering and interpreting diagnostic tests, performing certain procedures with appropriate training, optimizing patient status before endoscopic procedures, and developing individualized care plans.^1^ Many also lead specialized clinics, such as those focused on hepatitis C, metabolic dysfunction-associated steatotic liver disease, and obesity medicine, helping improve access to subspecialty care (Fig. 1).

Their impact extends to complex clinical decision making such as coordinating multidisciplinary care for high-risk patients undergoing endoscopic procedures. In these scenarios, APPs collaborate across specialties, working with oncologists, interventional radiologists, surgeons, hematologists, and primary care providers to help mitigate risk and ensure delivery of timely treatment.

### Procedural and periprocedural support

In procedural settings, APPs contribute meaningfully to pre- and postprocedure care. They perform preprocedure assessments, optimize patients for sedation, and mitigate bleeding and infection risks. Equally important, they educate patients about their procedures—clarifying expectations, addressing concerns, and improving compliance with preprocedure preparation. Postprocedure, they help interpret pathology results, initiate treatment plans, and ensure continuity of care through follow-up.

With proper training, APPs may also assist or perform ancillary procedures, such as motility studies, percutaneous endoscopic gastrostomy tube insertions, high-resolution anoscopy, and hemorrhoid banding. Their procedural support improves patient safety, operational efficiency, and the overall care experience.

### Patient education and advocacy

APPs play a pivotal role in educating patients about chronic disease management, lifestyle changes, medication adherence, and follow-up care. Their ability to build trust and improve health literacy is particularly valuable among underserved populations and those with limited access to subspecialty care.

### Leadership, education, research, and quality improvement

Beyond clinical care, APPs in gastroenterology are increasingly involved in leadership, education, clinical research, and quality improvement initiatives. Many lead or participate in quality improvement projects, serve on clinical committees, and contribute to academic research and clinical trials. Their involvement extends beyond their local institutions as more APPs are taking on active roles within national professional societies such as serving on (and chairing) committees. These roles not only help advance the profession but also ensure the APP perspective is represented in shaping the future of gastroenterology on a broader scale.

### Challenges and opportunities

The integration of APPs into gastroenterology practices has been shown to reduce wait times, enhance continuity of care, and support cost-effective care delivery.^2^ Their presence allows gastroenterology physicians to focus on complex cases and procedures. However, appropriate onboarding and ongoing training and mentorship are crucial to their success, both for new graduate APPs and those transitioning into gastroenterology from another specialty.

To thrive professionally, APPs need clearly defined roles, collaborative support (and mentorship) from physician colleagues, and the opportunity to practice at the top of their scope as defined by state and institutional guidelines. Inclusion and recognition are also essential—not only for effective team dynamics but also for mitigating burnout and promoting long-term career satisfaction.^3^

### Celebrating APP week

National APP Week, celebrated annually in September, is a time to recognize the invaluable contributions of NPs and PAs across all specialties. This year's theme, “Racing Toward the Future: Celebrating APPs,” underscores the forward momentum of the APP profession and its pivotal role in advancing care. Whether managing patients with decompensated cirrhosis, coordinating care for endoscopic resection of early gastrointestinal cancers, or supporting newly diagnosed patients with inflammatory bowel disease through complex treatment pathways, APPs are at the forefront of transformative gastroenterology care.

**Figure 2:**
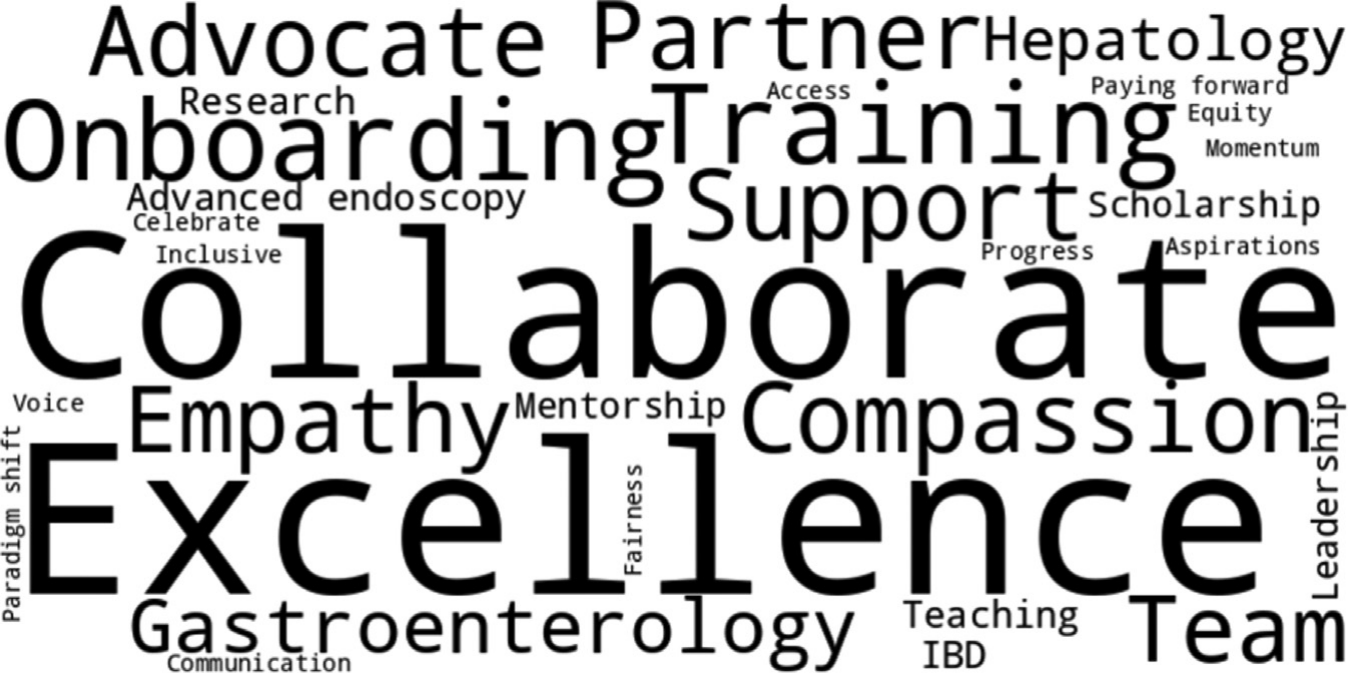
Core values and contributions of advanced practice providers in gastroenterology.

As gastroenterology continues to grow and evolve, APPs will remain central to shaping its future. This National APP Week, we honor their clinical excellence, leadership, and unwavering commitment to compassionate, collaborative, and patient-centered care (Fig. 2).

## Patient Consent

Informed consent was obtained from the patients for the publication of their information and imaging.

## Disclosure

All authors disclosed no financial relationships.
